# Spectrum of lower respiratory tract infections and antimicrobial resistance pattern in head and neck cancer patients undergoing chemoradiation

**DOI:** 10.12688/f1000research.162892.2

**Published:** 2025-11-15

**Authors:** Ashima Palia, Vaishnavi Singh, Suchitra Shenoy, Prerana Baruah, Pooja Prakash, Athiyamaan MS, Sourjya Banerjee, Johan Sunny, Paul Simon, Challapalli Srinivas, Dilson Lobo, Mamtha Suvarna, Abhishek Krishna

**Affiliations:** 1 Kasturba Medical College Mangalore, Manipal Academy of Higher Education, Karnataka, Manipal, 576 104, India; 2 Department of Microbiology, Kasturba Medical College Mangalore, Manipal Academy of Higher Education, Karnataka, Manipal, 576 104, India; 3 Department of Radiation Oncology, Kasturba Medical College Mangalore, Manipal Academy of Higher Education, Karnataka, Manipal, 576 104, India

**Keywords:** Head and Neck Neoplasms, Chemoradiotherapy, Lower Respiratory Tract Infections, Pseudomonas aeruginosa, Klebsiella pneumoniae, Antimicrobial Resistance, Multidrug-Resistant Bacteria, Microbiological Profile, Risk Factors, Empirical Antibiotic Therapy

## Abstract

**Background:**

Head and neck cancer (HNCs) is a major health issue worldwide, and India has contributed to approximately 2.4 lakh new cases in 2022. Definitive chemoradiation is the standard treatment for locally advanced disease but carries a risk of lower respiratory tract infections (LRTI) that add to morbidity, hospitalization, cost, and possible delay in treatment. The increasing incidence of antimicrobial resistance (AMR) has also contributed to management burden. This study aimed to assess the microbiological profiles and antimicrobial resistance patterns of lower respiratory tract infections in patients with head and neck cancer receiving chemoradiation.

**Methods:**

Patients who underwent definitive radiotherapy with or without chemotherapy and who developed LRTIs were included in the study. Sputum and tracheostomy suction tip cultures were obtained and processed using standard microbiological techniques such as Gram staining, biochemical tests, and VITEK-2 automated systems. Antimicrobial susceptibility was tested according to the Clinical and Laboratory Standards Institute (CLSI) and EUCAST recommendations. Clinical and treatment-related factors were documented and compared using SPSS version 23.0, with descriptive statistics, chi-square tests, t-tests, ANOVA, and logistic regression models.

**Results:**

Pseudomonas aeruginosa was the most frequently isolated pathogen (35.0%), followed by Klebsiella pneumoniae (16.7%), and Acinetobacter baumannii (10.0%). The pathogens were strongly resistant to fluoroquinolones and third-generation cephalosporins but were susceptible to carbapenems and aminoglycosides in the majority of isolates. Pseudomonas aeruginosa was the most frequent pathogen in all age groups and chemotherapy regimens (p<0.001).

**Conclusion:**

LRTIs in patients with HNC treated with chemoradiation were mainly caused by multidrug-resistant Pseudomonas aeruginosa and Klebsiella pneumoniae. Resistance patterns are crucial for directing empirical antibiotic therapy, minimizing treatment delays, and enhancing clinical outcomes.

## Introduction

Head and Neck Cancers (HNCs) cause a significant burden on global health, with India contributing substantially. In 2022, an estimated 2.4 lakh new cases of head and neck cancer were diagnosed in India. ^1^ The high incidence of HNCs in India is attributed to the use of tobacco, betel nut chewing, alcohol, and environmental factors such as exposure to pollution. ^2, 3^

The management of head and neck cancer is often a multimodal treatment that includes surgery, radiotherapy, chemotherapy, immunotherapy, or a combination of the three. ^4^ Of these, definitive chemoradiation therapy has been the standard of care for the treatment of locally advanced and some early stage head and neck cancers. ^5^ One of the major complications of this treatment is lower respiratory tract infections (LRTIs), which are common in immunocompromised populations. ^6^ The immunosuppressive action of chemotherapy and mucosal defense disruption with radiotherapy increase the vulnerability of patients to pulmonary infection. ^7^ Dysphagia can also lead to aspiration, thus increasing the risk of respiratory complications including pneumonia. ^8^ LRTIs in patients undergoing chemoradiation for head and neck cancer are a major cause of morbidity, leading to prolonged hospital stays, increased healthcare costs, and delays in the completion of treatment. ^6^

Studies have reported an incidence of infection of 12–19% in patients undergoing chemoradiotherapy for HNCs. ^6, 9^ In a large cohort of 5674 head and neck cancer patients, 238 bloodstream infections were reported, with increasing age, advanced stage, and poor performance status as key risk factors, while *S. aureus* was the predominant early pathogen and BSIs significantly contributed to early non-cancer mortality. ^10^

Infections during chemoradiation in patients with head and neck cancer contribute significantly to increased morbidity, financial toxicity, and treatment interruptions, which ultimately compromise oncologic outcomes. Treatment-related infections, most notably pneumonia, have been shown to prolong hospital stays and increase healthcare expenditures by as much as US$17,000 per episode, thereby amplifying the overall financial burden on patients. ^11^ Moreover, these adverse events intensify out-of-pocket expenses and potential loss of income, which are associated with a poorer quality of life and reduced treatment compliance. ^12^ In addition, infectious complications often necessitate unplanned treatment gaps. Studies indicate that interruptions in radiotherapy exceeding five days can reduce locoregional control rates and diminish overall survival. ^13^

Although infections are a major cause of morbidity and mortality, the effects of these infections are further aggravated by the increasing trend in antimicrobial resistance (AMR). The increase in antimicrobial resistance (AMR) has added to the problems of infection control in patients with cancer. Hospitalized cancer patients have 1.5–2 times higher antimicrobial resistance rates than non-cancer patients do. ^14, 15^ Widespread and inappropriate use of antibiotics in both inpatient and outpatient settings has resulted in an increase in the number of drug-resistant organisms. As a result, infections that were once manageable with routine antibiotics became harder to treat, leading to longer hospital stays and the need for costly and higher-end antibiotics. ^16^

The aim of this study was to evaluate an essential but poorly researched aspect of head and neck cancer (HNC) treatment, lower respiratory tract infections (LRTIs), in patients undergoing chemoradiation. While it is commonly thought that such patients are at an increased risk of infection due to factors such as immunosuppression, disruption of the mucosal barrier, and aspiration, there is a significant knowledge gap about the microbial epidemiology, predictors of risk of infection, and the emerging resistance patterns of the responsible pathogens. Understanding these factors is essential to maximize infection prevention strategies, guide empirical antibiotic therapy, and enhance clinical outcomes. By systematically analyzing the microbiological profile and resistance trends, this study aimed to provide valuable insights that can help clinicians make informed decisions, reduce treatment delays, and ultimately enhance the quality of care for HNC patients.

## Methods

### Study design and population

This study aimed to evaluate the spectrum of antimicrobial resistance and antibiotic sensitivity patterns in patients with head and neck cancer undergoing definitive radiation therapy, with or without chemotherapy, who developed lower respiratory tract infections (LRTIs) during treatment. The study had an ambispective study design, with data collected retrospectively from medical records and prospectively. Patients aged > 18 years who received radiation with curative intent were included. Patients treated with palliative intent, incomplete records, underlying immunosuppressive illnesses such as HIV/AIDS, organ transplantation, or chronic immunosuppressive treatments were excluded.

In our study, lower respiratory tract infections (LRTIs) were diagnosed using a combination of clinical, radiological, and microbiological criteria to ensure diagnostic accuracy. Clinically, patients presented with symptoms such as fever, cough with expectoration, dyspnea, and an increased respiratory rate. Radiological evaluation, including chest X-rays or CT scans, revealed findings consistent with pneumonia or bronchopulmonary infection. Microbiological confirmation was obtained through positive sputum cultures showing significant bacterial growth from validated sputum samples, thereby corroborating the clinical and imaging findings. Sputum culture and sensitivity tests were performed for bacterial pathogen identification in these patients. Suction tip cultures were obtained from the tracheostomy patients for microbiological examination.

### Sample collection and handling

To ensure that bacterial isolates and resistance patterns were not influenced by prior treatment, sputum samples were collected only from patients who had not yet been started on antibiotics. For suspected lower respiratory tract infections, early morning sputum samples were obtained in sterile containers with aseptic precautions. In patients for whom spontaneous expectoration of sputum was not possible, saline nebulization was used to aid sputum collection. In patients who underwent tracheostomy, suction-tip cultures were obtained as specimens for analysis. All specimens were sent within one hour of collection to the microbiology laboratory to enable maximum recovery.

To ensure sample quality, sputum samples were validated using Gram stain criteria, including only those with <10 epithelial cells and >25 polymorphonuclear leukocytes per low-power field. Accepted samples were cultured in a routine aerobic culture and incubated at 37°C for 24–48 h under suitable atmospheric conditions. Bacterial isolates were typed using routine biochemical tests, including catalase, oxidase, coagulase, and carbohydrate fermentation tests. Automated systems, such as VITEK®2 (bioMérieux, Inc), were employed for correct species-level identification.

### Antimicrobial susceptibility testing

Antibiotic sensitivity testing was performed using the Kirby-Bauer disk diffusion and VITEK 2 systems. The results were interpreted and reported based on the standards of the Clinical and Laboratory Standards Institute (CLSI) and European Committee on Antimicrobial Susceptibility Testing (EUCAST) guidelines. ^17, 18^ The bacterial isolates were tested against a panel of commonly used antimicrobial agents, including beta-lactams (penicillin, cephalosporins, and carbapenems), fluoroquinolones, aminoglycosides, and other classes suitable for the identified pathogen. The sensitivity results were reported as sensitive (S), intermediate (I), or resistant (R) based on the CLSI 2023 guidelines. ^17^

### Assessed clinical and therapeutic parameters

Demographic information, such as age and sex, and appropriate comorbid diseases, such as diabetes mellitus, hypertension, and COPD, were collected. The time gap between the initiation of treatment and the development of LRTI was recorded. Tumor-related characteristics such as location of the primary tumor, stage as per TNM staging system, and the treatment received, including radiotherapy parameters (i.e., total dose, fractionation schedule, and treatment duration) were noted. Chemotherapy-related characteristics, such as the type of agents administered and the total number of courses of chemotherapy administered, were documented.

### Statistical analysis

All statistical analyses were performed using SPSS version 23.0. ^19^ Descriptive and inferential statistical methods were used to examine the trends, associations, and resistance patterns among the bacterial isolates.

Categorical variables were reported as frequencies and proportions. Continuous variables are reported as mean values with corresponding standard deviations (SDs). The range of bacterial isolates from sputum and tracheal aspirate cultures was compared, and the most prevalent pathogens were identified. Antibiotic resistance patterns were ascertained by classifying bacterial isolates into sensitive (S), intermediate (I), and resistant (R) groups, and the proportion of resistant isolates for each of the tested antibiotics was approximated. Chi-square tests (χ ^2^) were used to examine categorical variables such as the distribution of bacterial species according to age group, sex, chemotherapy regimen, and radiation dose. Independent t-tests or Mann-Whitney U tests were used to compare continuous variables, such as age and days of treatment, between non-infected and infected patients. One-way ANOVA was employed to compare several group means of infection rates and antibiotic resistance patterns. To determine independent risk factors for LRTI development, a logistic model was developed to include age, sex, chemotherapy regimen, radiation dose, and timing of onset of infection. Statistical significance was determined at a p-value <0.05.

## Results

In total, 120 patients were included in the analysis. The median age of the study population was 54.2 years. The study population was predominantly male (75.0%), with females accounting for only 25.0%. The most commonly administered radiotherapy dose was 70 Gy (45.0%), followed by 66 Gy (35.0%) and 60 Gy (20.0%). Cisplatin was the most frequently used chemotherapeutic drug (60.8%). Most patients (83.3%) received between 1-5 cycles of chemotherapy, while only 6.7% underwent more than five cycles. The sputum was the primary specimen collected (90.0%), with suction tip samples in 10.0%. More than half of the specimens (52.5%) were collected between 15-22 days after treatment initiation. The detailed patient characteristics are shown in Table 1.

**Table 1.** Baseline patient characteristics.

Base line data		Number	Percentage
Age	18-37 years	8	6.7
	38-57 years	67	55.8
	58-77 years	41	34.2
	78 years and above	4	3.3
Gender	Female	30	25.0
	Male	90	75.0
Radio therapy dose	60 Gy	24	20.0
	66 Gy	42	35.0
	70 Gy	54	45.0
Chemotherapy used	Carboplatin	12	10.0
	Cisplatin	73	60.8
	Cisplatin + Capecitabine	23	19.2
	No Chemotherapy	12	10.0
Number of chemotherapy cycle	0	12	10.0
	1-5 cycle	100	83.3
	>5 cycle	8	6.7
Specimen type	Sputum	108	90.0
	Suction Tip	12	10.0
Days between start of treatment and sputum collection	7-14 days	21	17.5
	15-22 days	63	52.5
	23-30 days	32	26.7
	31-38 days	4	3.3

The most commonly isolated organisms were Pseudomonas aeruginosa (35.0%),Klebsiella pneumoniae (16.7%), Acinetobacter baumannii (10.0%), Staphylococcus aureus (9.2%), Escherichia coli (6.7%), Haemophilus influenzae, and Streptococcus pneumoniae (5.0% each) ( Table 2).

**Table 2.** Details of organisms isolated.

Organisms	Frequency	Percentage
Achromobacter Xylosoxidans	2	1.7
Acinetobacter Baumannii	12	10.0
Acinetobacter lwoffii	2	1.7
Burkholderia Cepacia	1	.8
Citrobacter Koseri	1	.8
Corynebacterium Striatum	1	.8
Diphtheroid species	2	1.7
Enterobacter Aerogenes	1	.8
Enterobacter species	1	.8
Escherichia Coli	8	6.7
Haemophilus Influenzae	6	5.0
Klebsiella Pneumoniae	20	16.7
Moraxella species	1	.8
Proteus Vulgaris	1	.8
Pseudomonas Aeruginosa	42	35.0
Staphylococcus Aureus	11	9.2
Stenotrophomonas Maltophilia	2	1.7
Streptococcus Pneumoniae	6	5.0

Klebsiella pneumoniae showed the highest resistance to ampicillin (40%), ceftriaxone (30%), and ciprofloxacin (25%), limiting their effectiveness. In contrast, amikacin (100%), meropenem (95%), Cefoperazone+Sulbactam, Gentamicin, and Imipenem (90%) exhibited high sensitivity, making them preferred treatment options.

Pseudomonas aeruginosa showed the highest resistance to levofloxacin (33.3%), followed by trimethoprim/sulfamethoxazole (26.2%) and ciprofloxacin (26.2%). High sensitivities were observed for Cefoperazone+Sulbactam (81.0%), imipenem (78.6%), ceftazidime (76.2%), and meropenem (73.8%). Intermediate susceptibility to cefepime (14.3%), minocycline (7.1%), and Ticarcillin/Clavulanic Acid (7.1%) was noted.

Among Acinetobacter baumannii isolates, the highest resistance was observed with Piperacillin+Tazobactam (33.3%), cefepime resistance was also notable at 25.0%, although 75.0% of isolates remained sensitive, ciprofloxacin exhibited a 25.0% resistance rate, and trimethoprim/sulfamethoxazole showed 25.0% resistance. Among Escherichia coli, ceftriaxone showed the highest resistance (62.5%), followed by ampicillin (50.0%). Ciprofloxacin showed 50.0% resistance with 25.0% intermediate sensitivity, while trimethoprim/sulfamethoxazole exhibited 37.5% resistance. For H influenzae, cotrimoxazole showed the highest resistance (66.7%), followed by ampicillin (50.0%). Cefuroxime and Ciprofloxacin both showed 33.3% resistance, while Amoxycillin/Clavulanic Acid showed 16.7% resistance. In contrast, azithromycin (100%) and cefotaxime (83.3%) showed the highest sensitivities, making them the preferred treatment options. Levofloxacin, Meropenem, and Cefuroxime were effective (66.7% sensitivity).

The resistance rates to higher-end antibiotics revealed that Piperacillin+Tazobactam exhibited the highest resistance at 15.0% (18 cases), followed by imipenem at 10.0% (12 cases). Cefoperazone+ sulbactam showed a resistance rate of 5.8% (seven cases), while tigecycline had the lowest resistance at 1.7% (two cases) ( Table 3).

**Table 3.** Resistance rates for higher end antibiotics.

Antibiotics	Frequency	Percentage
Cefoperazone+Sulbactum	7	5.8
Imipenem	12	10.0
Piperacillin+Tazobactam	18	15.0
Tigecycline	2	1.7

### Organisms and age

**Figure 1.**
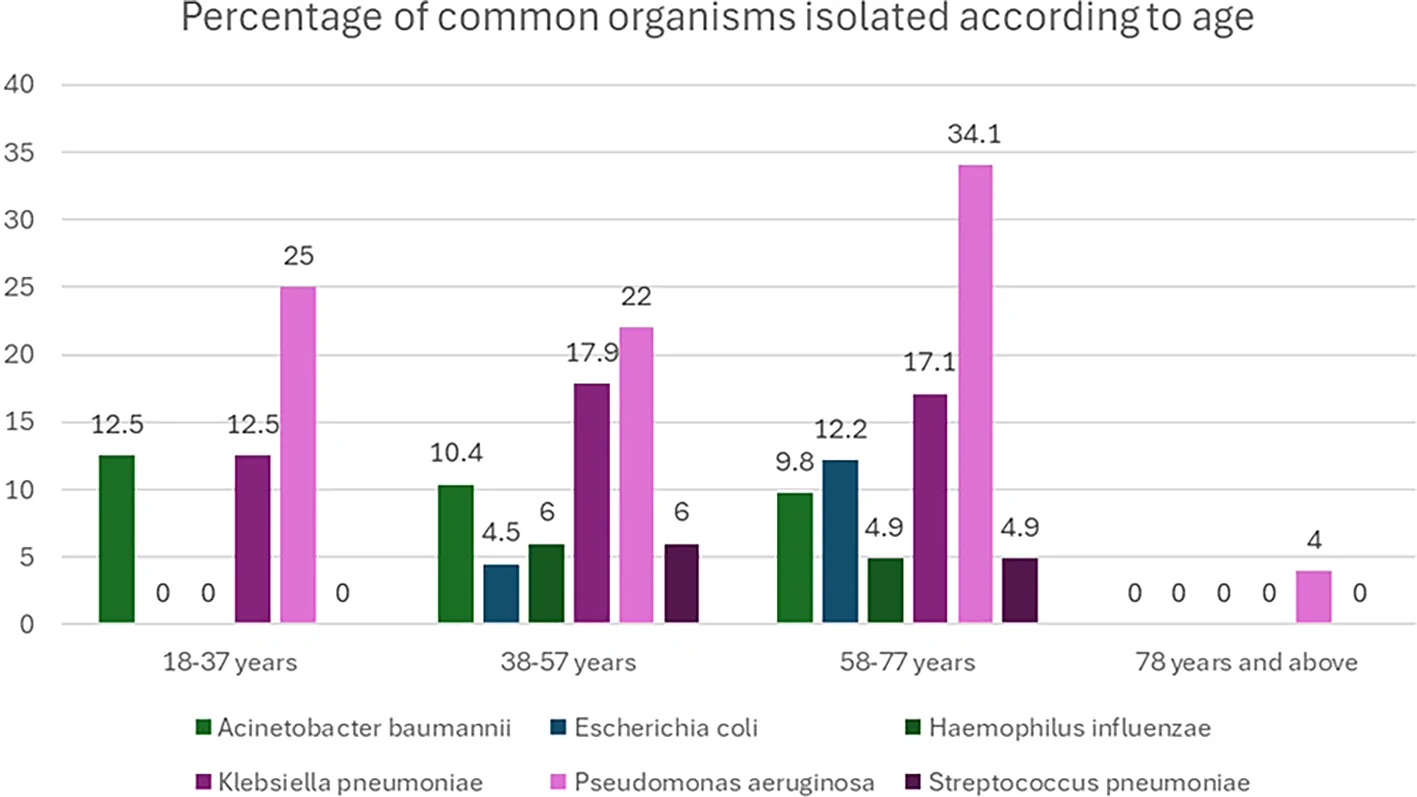
Percentage of common organisms isolated according to age. (The figure is created by the authors based on original data and hence does not require permission for reproduction).

Although not statistically significant in the 18-37 years age group, the most common organisms were Pseudomonas aeruginosa and Staphylococcus aureus, both found in 25.0% of the cases (p=0.36). In the 38-57 years age group, Pseudomonas aeruginosa was the most prevalent organism, detected in 32.8% of cases, followed by Klebsiella pneumoniae (17.9%) (p=0.44) For the 58-77 years age group, Pseudomonas aeruginosa remained the most common organism (34.1%), followed by Klebsiella pneumoniae (17.1%) (p=0.56). Pseudomonas aeruginosa was the only organism detected in the 78 years and above category, accounting for 100% of the cases ( Figure 1).

### Organism and onset of LRTI

**Figure 2.**
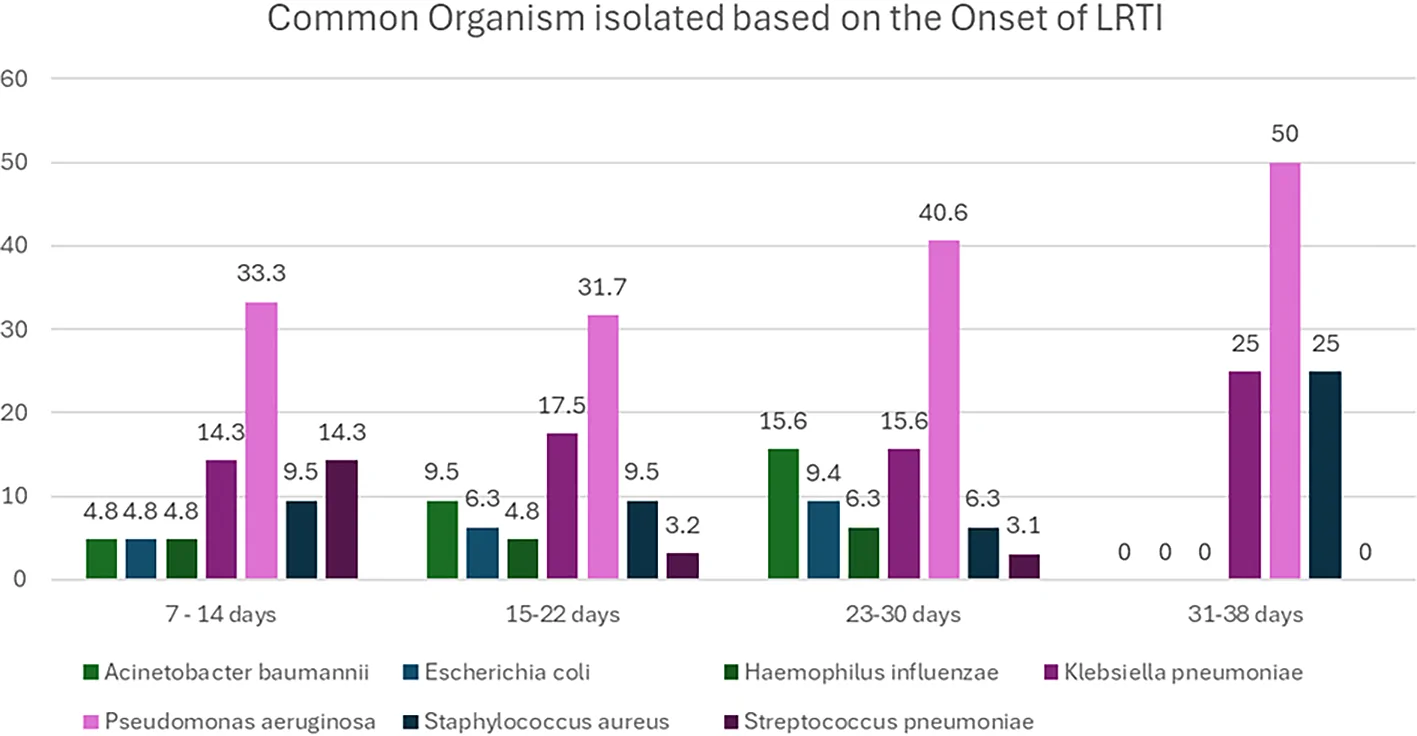
Common Organisms isolated based on the onset of LRTI. (The figure is created by the authors based on original data and hence does not require permission for reproduction).

Between 7-14 days, Pseudomonas aeruginosa (33.3%) was the most common isolate, followed by Klebsiella pneumoniae (14.3%), Streptococcus pneumoniae (14.3%), and Staphylococcus aureus (9.5%) (p <0.001). In 15-22 days, Pseudomonas aeruginosa (31.7%) remained dominant, followed by Klebsiella pneumoniae (17.5%), Staphylococcus aureus (9.5%), and Escherichia coli (6.3%). By 23-30 days, Pseudomonas aeruginosa (40.6%) increased, while Klebsiella pneumoniae (15.6%) and Acinetobacter baumannii (15.6%) increased significantly (p<0.001). In 31-38 days, Pseudomonas aeruginosa (50.0%) was the highest, followed by Klebsiella pneumoniae (25.0%) and Staphylococcus aureus (25.0%) (p=0.34) ( Figure 2).

### Organism and chemotherapy

Based on the chemotherapy regimen used, the most frequently isolated organisms varied across the groups. In the Carboplatin group, Klebsiella pneumoniae (50.0%) was the most common, followed by Haemophilus influenzae (16.7%) and Pseudomonas aeruginosa (16.7%) (p<0.001) The Cisplatin group had a high prevalence of Pseudomonas aeruginosa (37.0%), Escherichia coli (8.2%), Klebsiella pneumoniae (11.0%), and Staphylococcus aureus (11.0%) (p=0.04). Among patients receiving Cisplatin + Capecitabine, Pseudomonas aeruginosa (43.5%) was predominant, followed by Klebsiella pneumoniae (17.4%), and Acinetobacter baumannii (13.0%) (p=0.03). In the group that did not receive chemotherapy, Acinetobacter baumannii (25.0%) and Pseudomonas aeruginosa (25.0%) were the most frequently detected, followed by Escherichia coli (16.7%) and Streptococcus pneumoniae (16.7%) (p=0.23).

## Discussion

Respiratory infections are a significant complication in patients undergoing chemoradiation therapy for head and neck cancers, given their immunocompromised status, both from the disease itself and its treatment. This article provides a spectrum of antimicrobial resistance and susceptibility patterns in these patients, which is of critical significance in their management.

The temporal pattern of infections indicated that Pseudomonas aeruginosa prevailed in all periods observed consistently, increasing from 33.3% in the first phase (7-14 days) to 50.0% in later phases (31-38 days). This pattern can be considered an indication of the pathogen’s ability to survive in healthcare settings and as a causative agent of infections later on. ^20^ The presence of Acinetobacter baumannii and Klebsiella pneumoniae in the 23-30 days group might indicate the occurrence of secondary infections, probably as a consequence of prolonged hospitalization, mechanical ventilation, and the effect of antibiotic treatment. ^21^

The higher frequency of Pseudomonas aeruginosa (35.0%), the most frequently isolated pathogen, is in agreement with previous studies that demonstrated immunocompromised patients, particularly those undergoing chemoradiotherapy, to be highly vulnerable to infection by opportunistic pathogens. ^22, 23^ Pseudomonas aeruginosa is characterized by inherent resistance mechanisms and the ability to develop further resistance; thus, it is prevalent in this population. The higher rates of Klebsiella pneumoniae (16.7%) and Acinetobacter baumannii (10.0%) further indicate that such patients are highly vulnerable to hospital-acquired infections and ventilation-related infections.

Several factors determine observed microbial diversity and resistance patterns. Patients undergoing chemoradiotherapy tend to develop mucositis, xerostomia, and impaired mucosal immunity, favoring the colonization and invasion of bacteria in the lower respiratory tract. ^5, 6^ The widespread use of broad-spectrum antibiotics in these patients has the potential to induce antimicrobial resistance. Antimicrobial resistance (AMR) is a significant issue in oncology, particularly in patients with head and neck cancer (HNC) undergoing chemoradiation therapy. AMR occurs when bacterial pathogens evolve to resist the action of antibiotics, which ultimately renders traditional treatment ineffective.

The implications of AMR in oncology are dire. Studies have proven a direct link between resistant bacterial infections and increased mortality in cancer patients, particularly those with hematologic malignancies or solid tumors undergoing immunosuppressive therapy. ^24, 25^ The overuse and misuse of antibiotics, typically triggered by empirical treatment strategies in critically ill cancer patients, are reasons for the rapid emergence of resistant strains. ^26^ The antimicrobial resistance pattern observed in this study is alarming in relation to the effectiveness of commonly used antibiotics. The high resistance of Klebsiella pneumoniae to ampicillin (40%) and ceftriaxone (30%) indicates that beta-lactam antibiotics may not be effective first-line treatment strategies. Similarly, Pseudomonas aeruginosa was highly resistant to levofloxacin (33.3%) and ciprofloxacin (26.2%), most likely because of the widespread empirical use of fluoroquinolones. Conversely, higher sensitivity rates were observed with Amikacin, Meropenem, and Cefoperazone+Sulbactam, which suggests their potential use in the treatment of infections in this patient population.

Radiotherapy is one of the areas of highest importance where treatment gaps can have negative impacts on outcomes. Infection- or treatment-related toxicity-induced delays in chemoradiation have been linked to worse survival outcomes and reduced therapeutic efficacies. Evidence indicates that even brief treatment delays of more than five days may lead to reduced tumor control probabilities and overall survival. ^27, 28^

Antibiotic-resistant infections require higher-end antibiotics, longer hospital stays, and intensive care unit hospitalizations, all of which add up to mounting healthcare expenditure. ^29^ In low- and middle-income nations, such as India, where the economic costs of treating cancer are already borne by the patient, the additional cost of more effective newer-generation antibiotics might make them unaffordable, resulting in suboptimal infection treatment and mortality. ^30, 31^ In addition, the indirect costs in terms of productivity loss and burden to caregivers add to the economic costs of the enrolled families.

Given the high prevalence of multidrug-resistant organisms in our study, a targeted approach to antimicrobial therapy is crucial. Implementing robust antibiotic stewardship programs is essential for mitigating the spread of resistant pathogens. ^32^ Strategies such as de-escalation based on culture results, restriction of broad-spectrum antibiotics to necessary cases, and rotation of empirical regimens can help to preserve antibiotic effectiveness. Additionally, infection control measures, including stringent hand hygiene, environmental sanitation, and surveillance of resistance patterns, play a critical role in curbing the transmission of multidrug-resistant organisms in oncology settings. Empirical antibiotic selection should be guided by local resistance patterns and periodic surveillance to ensure optimal treatment outcomes. Additionally, the development of new antimicrobial agents and combination therapies may play a vital role in managing infections in this vulnerable population.

This study had certain limitations. Although the sample size is reasonable, it may not be generalizable to the entire patient population with head and neck cancer undergoing chemoradiotherapy. Second, our study mainly used sputum samples, which may not always be generalizable to lower respiratory tract infections. Employing bronchoalveolar lavage or protected specimen brushing would provide more definitive microbiological results. Future studies using these methods in combination may provide a better understanding of the clinical relevance of antimicrobial resistance in this group of patients.

## Conclusion

Our study highlights the high prevalence of Pseudomonas aeruginosa and Klebsiella pneumoniae in respiratory infections among patients with head and neck cancer undergoing chemoradiotherapy, with significant resistance patterns to commonly used antibiotics. These findings underscore the importance of antibiotic stewardship, infection control measures, and periodic surveillance to optimize antimicrobial therapy and improve patient outcomes.

## Consent

Written informed consent to take part in the research has been obtained from all patients when indicated. Consent for publication is not applicable, as all data are anonymized and no images are being published.

## Ethics statement

This study was approved by the Institutional Ethics Committee of Kasturba Medical College, Mangalore, vide Protocol No.- IEC KMC MLR 12/2023/502 dated 21.12.2023. This study was conducted in accordance with the ethical guidelines and regulations set forth by the institution. The study was conducted in accordance with the principles of the Declaration of Helsinki.

## Author contributions

All authors contributed equally to the conceptualization, methodology, data collection, analysis, manuscript writing, and revision. All the authors have read and approved the final version of the manuscript.

## Data Availability

FIGSHARE: Spectrum of Lower Respiratory Tract Infections and antimicrobial resistance pattern in head and neck cancer patients undergoing chemoradiation. Dataset. https://doi.org/10.6084/m9.figshare.28533335.v1. ^33^ This project contains the following underlying data:
• Datasets.xlsx Data are available under the terms of the Creative Commons Zero “No rights reserved” data waiver (CC0 4.0 Public domain dedication).
