# Supplementary material for: Spectrum of Lower Respiratory Tract Infections and Antimicrobial Resistance Pattern in Head and Neck Cancer Patients Undergoing Chemoradiation
Source: F1000Res. 2026 Oct 12;14:314. Originally published 2025 Mar 20. [Version 3] doi: 10.12688/f1000research.162892.3 (PMC13550927; doi:10.12688/f1000research.162892.3)
Supplement: Peer review history

# Reviewer report: Reviewer response for version 3

**DOI:** 10.5256/f1000research.212194.r529344

**Published:** 2026-11-02

**Authors:** Jiankang Zhao

**Affiliations:** 1 China-Japan Friendship Hospital, Beijing, China

No more comments.

Is the work clearly and accurately presented and does it cite the current literature?

Partly

If applicable, is the statistical analysis and its interpretation appropriate?

Yes

Are all the source data underlying the results available to ensure full reproducibility?

Yes

Is the study design appropriate and is the work technically sound?

Partly

Are the conclusions drawn adequately supported by the results?

Yes

Are sufficient details of methods and analysis provided to allow replication by others?

Partly

Reviewer Expertise:

Infection

I confirm that I have read this submission and believe that I have an appropriate level of expertise to confirm that it is of an acceptable scientific standard.

# Reviewer report: Reviewer response for version 2

**DOI:** 10.5256/f1000research.190184.r497728

**Published:** 2026-07-11

**Authors:** Saurabha Kumar

**Affiliations:** 1 Narayana Health City, Bangalore, Karnataka, India

This is a unique study looking into the microbial pattern and AMR resistance patterns among patients receiving Concurrent chemoradiotherapy in head and neck patients. The treatment is challenging and at the same time concomitant infections keep the physicians guessing since out-comes of cancer treatment can be affected if radiotherapy treatment breaks would be required. Such studies give an insight to the patterns of infection and current AMR patterns and help in treatment decision making.

Is the work clearly and accurately presented and does it cite the current literature?

Yes

If applicable, is the statistical analysis and its interpretation appropriate?

Yes

Are all the source data underlying the results available to ensure full reproducibility?

Yes

Is the study design appropriate and is the work technically sound?

Yes

Are the conclusions drawn adequately supported by the results?

Yes

Are sufficient details of methods and analysis provided to allow replication by others?

Yes

Reviewer Expertise:

Radiation Oncology with special intrest in Head and neck cancers.

I confirm that I have read this submission and believe that I have an appropriate level of expertise to confirm that it is of an acceptable scientific standard.

# Reviewer report: Reviewer response for version 2

**DOI:** 10.5256/f1000research.190184.r497731

**Published:** 2026-07-02

**Authors:** Lanisha Sequeira

**Affiliations:** 1 Father Muller Medical College, Mangalore, Karnataka, India

This is an important article in the present era where we are observing significant antimicrobial resistance pattern in cancer care. I congratulate the authors for taking up the study.

 Methodology:

 In the version 1, the methodology of sputum collection and processing was inadequate but the authors have elaborated it in the version 2.

 Discussion

 Certain antibiotics have not been tested. One reason could be due to institutional and regional incidence of the microbes. The authors if needed can clarify on that.

Is the work clearly and accurately presented and does it cite the current literature?

Yes

If applicable, is the statistical analysis and its interpretation appropriate?

Yes

Are all the source data underlying the results available to ensure full reproducibility?

Yes

Is the study design appropriate and is the work technically sound?

Yes

Are the conclusions drawn adequately supported by the results?

Yes

Are sufficient details of methods and analysis provided to allow replication by others?

Yes

Reviewer Expertise:

Oncology, Head and Neck Cancer, Breast Cancer

I confirm that I have read this submission and believe that I have an appropriate level of expertise to confirm that it is of an acceptable scientific standard.

# Reviewer report: Reviewer response for version 1

**DOI:** 10.5256/f1000research.179163.r373468

**Published:** 2025-04-02

**Authors:** Jiankang Zhao

**Affiliations:** 1 China-Japan Friendship Hospital, Beijing, China

Summary of the Article 

 This study investigates the microbiological profile and antimicrobial resistance (AMR) patterns of lower respiratory tract infections (LRTIs) in head and neck cancer (HNC) patients undergoing chemoradiation. The authors identified Pseudomonas aeruginosa (35%) and Klebsiella pneumoniae (16.7%) as the predominant pathogens, with high resistance to fluoroquinolones and third-generation cephalosporins but susceptibility to carbapenems and aminoglycosides. The ambispective design included 120 patients, with sputum/tracheal suction cultures analyzed using standard microbiological methods and automated systems (VITEK-2). Statistical analyses (chi-square, t-tests, ANOVA, logistic regression) were performed to correlate clinical factors with infection patterns.

 1. Is the work clearly and accurately presented and does it cite the current literature? 

    Partly 

    - Strengths: The introduction contextualizes the burden of HNC in India and highlights the clinical significance of LRTIs in chemoradiation patients. References to AMR trends in cancer patients are relevant. 

    - Criticisms:  The introduction does not cite recent studies (post-2020) on AMR patterns in HNC patients, particularly in Indian cohorts.  Comparisons to similar studies would strengthen the rationale.

 2. Is the study design appropriate and is the work technically sound? 

    Partly 

    - Strengths: The design in this sudy is suitable for evaluating microbiological profiles. Standard microbiological methods (Gram staining, VITEK-2) and CLSI/EUCAST guidelines ensure technical validity. 

    - Criticisms:  The study does not clarify how sputum samples were validated for quality to exclude oral flora contamination. Comorbidities (e.g., diabetes, COPD) and prior antibiotic use (a critical driver of AMR) are not analyzed as covariates in the logistic regression model. 

 3. Are sufficient details of methods and analysis provided to allow replication by others? 

    Partly 

    - Strengths: The microbiological protocols (culture, biochemical tests, VITEK-2) and statistical tests are described. 

    - Criticisms:  The criteria for diagnosing LRTIs (e.g., clinical vs. radiological vs. microbiological confirmation) are not explicitly defined.

Is the work clearly and accurately presented and does it cite the current literature?

Partly

If applicable, is the statistical analysis and its interpretation appropriate?

Yes

Are all the source data underlying the results available to ensure full reproducibility?

Yes

Is the study design appropriate and is the work technically sound?

Partly

Are the conclusions drawn adequately supported by the results?

Yes

Are sufficient details of methods and analysis provided to allow replication by others?

Partly

Reviewer Expertise:

lower respiratory tract infections, virulence and resistance of bacteria

I confirm that I have read this submission and believe that I have an appropriate level of expertise to confirm that it is of an acceptable scientific standard, however I have significant reservations, as outlined above.

# Response

**Published:** 2025-04-04

**Authors:** Abhishek Krishna

**Affiliations:** Radiation Oncology, Manipal Academy of Higher Education, Mangalore, Karnataka, India

1. Is the work clearly and accurately presented and does it cite the current literature?

    Partly

    - Strengths: The introduction contextualizes the burden of HNC in India and highlights the clinical significance of LRTIs in chemoradiation patients. References to AMR trends in cancer patients are relevant.

    - Criticisms:  The introduction does not cite recent studies (post-2020) on AMR patterns in HNC patients, particularly in Indian cohorts.  Comparisons to similar studies would strengthen the rationale.

**Authors Reply: **We acknowledge the need to incorporate recent studies on antimicrobial resistance (AMR) patterns in head and neck cancer patients, particularly from Indian cohorts. We will revise the introduction to include relevant post-2020 studies comparing our findings with similar research in the revised manuscript.

 2. Is the study design appropriate and is the work technically sound?

    Partly

    - Strengths: The design in this sudy is suitable for evaluating microbiological profiles. Standard microbiological methods (Gram staining, VITEK-2) and CLSI/EUCAST guidelines ensure technical validity.

    - Criticisms:  The study does not clarify how sputum samples were validated for quality to exclude oral flora contamination. Comorbidities (e.g., diabetes, COPD) and prior antibiotic use (a critical driver of AMR) are not analyzed as covariates in the logistic regression model.

**Authors Reply: **To ensure sample quality, we validated sputum samples using Gram stain criteria, including only those with <10 epithelial cells and >25 polymorphonuclear leukocytes per low-power field. We will  mention this in the methodology section for clarity.

 Regarding prior antibiotic use, we included only patients whose sputum samples were collected before initiating antibiotic therapy. This ensures that our bacterial isolates and resistance patterns were not influenced by prior treatment.

 As our study specifically focused on head and neck cancer patients undergoing chemoradiation, we did not analyze the correlation between comorbidities and lower respiratory tract infections (LRTI). However, we acknowledge the importance of this aspect and will clarify this limitation in the discussion section.

 3. Are sufficient details of methods and analysis provided to allow replication by others?

    Partly

    - Strengths: The microbiological protocols (culture, biochemical tests, VITEK-2) and statistical tests are described.

    - Criticisms:  The criteria for diagnosing LRTIs (e.g., clinical vs. radiological vs. microbiological confirmation) are not explicitly defined.

**Authors Reply:** We acknowledge the need to clearly define the criteria for diagnosing lower respiratory tract infections (LRTIs). In our study, LRTIs were diagnosed based on a combination of clinical, radiological, and microbiological criteria:

 Clinical Criteria: Presence of symptoms such as fever, cough with expectoration, dyspnea, and increased respiratory rate.

 Radiological Criteria: Chest X-ray or CT scan findings suggestive of pneumonia or bronchopulmonary infection.

 Microbiological Criteria: Positive sputum cultures with significant bacterial growth from validated sputum samples.

 We will  include these diagnostic criteria in the methodology section for clarity in the revised manuscript **.**

 Is the work clearly and accurately presented and does it cite the current literature?

 Partly

**Authors Reply: **We will include a few more recent references in the introduction part as said above in the revised manuscript.

# Response

**Published:** 2025-09-04

**Authors:** Abhishek Krishna

**Affiliations:** Radiation Oncology, Manipal Academy of Higher Education, Mangalore, Karnataka, India

Dear Sir, We have shared our comments few month back.
